# STRUMM: An Intelligent, Adaptive Software Package for Older Adults With a Cognitive Impairment

**DOI:** 10.1093/geroni/igab046.733

**Published:** 2021-12-17

**Authors:** Sara Czaja, Marco Ceruso, Walter Boot, Neil Charness, Wendy Rogers

**Affiliations:** 1 Weill Cornell Medicine/Center on Aging and Behavioral Research, New York, New York, United States; 2 Weill Cornell Med, New York, New York, United States; 3 Florida State University, Tallahassee, Florida, United States; 4 University of Illinois Urbana-Champaign, Champaign, Illinois, United States

## Abstract

Many older adults have a cognitive impairment (CI), which negatively impacts on their quality of life and threatens their independence. In this presentation, we provide an overview of the conceptual framework, structure, and processes of our multi-site Center, ENHANCE, which is focused on developing technology support for aging adults with a CI. ENHANCE has two cross-site research projects, two cross-site development projects, training, and dissemination components. A core battery of measures is collected across all projects. We also discuss the Supportive Technology Resources through Usability & Machine-learning Methods (STRUMM) research project, which focuses on an innovative intelligent adaptive software package aimed at providing cognitive and social support, and support for resource access to aging adults with a CI. STRUMM is designed to meet the user’s varying cognitive needs. Finally, we present preliminary data regarding the perceived usability and value of STRUMM from our clinical partners and potential user groups.

